# Decreased miR-497-5p Suppresses IL-6 Induced Atrophy in Muscle Cells

**DOI:** 10.3390/cells10123527

**Published:** 2021-12-14

**Authors:** Paula P. Freire, Sarah S. Cury, Letícia O. Lopes, Geysson J. Fernandez, Jianming Liu, Leonardo Nazario de Moraes, Grasieli de Oliveira, Jakeline S. Oliveira, Diogo de Moraes, Otavio Cabral-Marques, Maeli Dal-Pai-Silva, Xiaoyun Hu, Da-Zhi Wang, Robson F. Carvalho

**Affiliations:** 1 Department of Structural and Functional Biology, Institute of Biosciences, São Paulo State University, UNESP, Botucatu 18618-689, Brazil; freirepp2@gmail.com (P.P.F.); sarahscury@gmail.com (S.S.C.); leticia.oliveira@unesp.br (L.O.L.); papacriolla@gmail.com (G.J.F.); leonardonmunesp@gmail.com (L.N.d.M.); oliveira.grase@gmail.com (G.d.O.); jakeline.oliveira@unesp.br (J.S.O.); dioxiide2@gmail.com (D.d.M.); maeli.dal-pai@unesp.br (M.D.-P.-S.); 2 Department of Immunology, Institute of Biomedical Sciences, University of São Paulo, São Paulo 05508-000, Brazil; otavio.cmarques@gmail.com; 3 Faculty of Medicine, University of Antioquia, UdeA, Medellín 050010, Colombia; 4 Department of Cardiology, Boston Children’s Hospital, Harvard Medical School, Boston, MA 02115, USA; liu10ster@gmail.com (J.L.); xhu008@gmail.com (X.H.); dazhiw@usf.edu (D.-Z.W.); 5 Department of Clinical and Toxicological Analyses, School of Pharmaceutical Sciences, University of São Paulo, São Paulo 05508-000, Brazil; 6 Network of Immunity in Infection, Malignancy, and Autoimmunity (NIIMA), Universal Scientific Education and Research Network (USERN), São Paulo 05508-000, Brazil; 7 Harvard Stem Cell Institute, Harvard University, Cambridge, MA 02138, USA

**Keywords:** Interleukin-6, muscle wasting, microRNAs, inflammation

## Abstract

Interleukin-6 (IL-6) is a pro-inflammatory cytokine associated with skeletal muscle wasting in cancer cachexia. The control of gene expression by microRNAs (miRNAs) in muscle wasting involves the regulation of thousands of target transcripts. However, the miRNA-target networks associated with IL6-induced muscle atrophy remain to be characterized. Here, we show that IL-6 promotes the atrophy of C2C12 myotubes and changes the expression of 20 miRNAs (5 up-regulated and 15 down-regulated). Gene Ontology analysis of predicted miRNAs targets revealed post-transcriptional regulation of genes involved in cell differentiation, apoptosis, migration, and catabolic processes. Next, we performed a meta-analysis of miRNA-published data that identified miR-497-5p, a down-regulated miRNAs induced by IL-6, also down-regulated in other muscle-wasting conditions. We used miR-497-5p mimics and inhibitors to explore the function of miR-497-5p in C2C12 myoblasts and myotubes. We found that miR-497-5p can regulate the expression of the cell cycle genes *CcnD2* and *CcnE1* without affecting the rate of myoblast cellular proliferation. Notably, miR-497-5p mimics induced myotube atrophy and reduced *Insr* expression. Treatment with miR-497-5p inhibitors did not change the diameter of the myotubes but increased the expression of its target genes *Insr* and *Igf1r*. These genes are known to regulate skeletal muscle regeneration and hypertrophy via insulin-like growth factor pathway and were up-regulated in cachectic muscle samples. Our miRNA-regulated network analysis revealed a potential role for miR-497-5p during IL6-induced muscle cell atrophy and suggests that miR-497-5p is likely involved in a compensatory mechanism of muscle atrophy in response to IL-6.

## 1. Introduction

Cancer cachexia is a muscle wasting syndrome that affects ~50% of all cancer patients and is the direct cause of ~20–30% of cancer-related deaths [1,2,3]. The main problem related to cancer cachexia prevention is that patients are only diagnosed with cachexia when they lose 7–15% of body mass [4,5]. Radio- and chemotherapy treatments may aggravate cachexia progression [6] and diminish the quality of life due to a loss of skeletal muscle mass [7].

Chronic inflammation is a hallmark of cancer cachexia due to the release of the tumor and host-derived factors that induce muscle wasting [8]. Among these factors, proinflammatory cytokines such as interleukin-1β (IL-1β), tumor necrosis factor (TNF)-α, interferon (INF)-γ, and interleukin-6 (IL-6) play a crucial role in the development of muscle wasting [9,10,11,12], by acting both individually and synergistically in the loss of muscle proteins [13,14,15,16]. Specifically, the IL-6 cytokine is an essential player in the progression of cachexia in cancer as its levels correlate with the survival of advanced cancer patients [17], and it is a sensitive indicator of muscle mass loss in patients with colon and lung cancer [18,19,20]. Moreover, the overexpression of IL-6 induces loss of muscle mass in various models of cancer cachexia [18,21,22,23,24,25]. However, IL-6 has a pleiotropic role in skeletal muscle cells that ranges from promyogenic to atrophic [26]. For example, suramin, an antagonist of the IL-6 receptor, markedly decreased the rate of cachexia in mice with C26 tumors [22], while neutralizing antibodies against IL-6 decreased muscle wasting muscle in rats with cancer cachexia [23]. In addition, inhibition of IL-6 using soluble receptor antibody (sIL-6R) reduced muscle atrophy in a mouse model of intestinal cancer [27]. Although preclinical studies with IL-6 have shown some efficiency, there is no consensus about anti-IL 6 therapies in patients [28]. For example, cachectic patients with non-small cell lung cancer treated with an anti-IL-6 antibody ameliorated weight loss while decreasing anemia and anorexia [29,30]. However, other clinical trials have not shown improvement in clinical outcomes of IL-6 inhibitors in patients with multiple myeloma and prostate cancer [31,32].

Some recent studies have explored molecular mechanisms of IL-6-induced muscle atrophy [9,26,33,34]; however, the post-transcriptional regulation induced by IL-6 in muscle cells remains largely unknown. Among the post-transcriptional mechanisms, microRNAs (miRNAs)-based regulatory networks play an essential role in muscle-wasting conditions [35,36,37,38,39,40,41,42]. While these previous studies have identified distinct global miRNA transcriptional profiles in muscular atrophy, they demonstrate the need to exploit specific miRNAs-based regulatory networks associated with key inducers of muscle wasting. Here, we investigated the global miRNA expression profile in myotubes differentiated from C2C12 myoblasts treated with IL-6 to uncover new specific miRNAs-based regulatory networks associated with IL-6-induced muscle atrophy.

## 2. Material and Methods

### 2.1. Cell Culture and Myoblast Differentiation

C2C12 mouse myoblasts (ATCC^®^ CRL-1772^TM^) were cultured in a growth medium (GM) consisting of Dulbecco’s modified Eagle’s medium (DMEM, Thermo Fisher Scientific, Waltham, MA, USA) supplemented with 1% Penicillin–Streptomycin (Thermo Fisher Scientific, Waltham, MA, USA) and 10% fetal bovine serum (Thermo Fisher Scientific, Waltham, MA, USA), at 37 °C and 5% CO_2_. Near-confluent cells (80% to 90%) were induced to differentiate in a differentiation medium (DM), consisting of DMEM plus 2% horse serum (Thermo Fisher Scientific, Waltham, MA, USA) and 1% penicillin-streptomycin solution for five days. Three concentrations of mouse recombinant-IL-6 (10 ng/mL, 50 ng/mL, and 100 ng/mL) (PMC0065, Thermo Fisher Scientific, Waltham, MA, USA) were tested in differentiated myotubes to induce muscle wasting (Supplementary Figure S1A). All experiments were carried out using at least three independent replicates per group.

### 2.2. Oligonucleotides and Transfection

The mimic or inhibitor mmu-miR-497-5p miRIDIAN microRNAs (mimic, C310724-01-0002; hairpin inhibitor, IH310724-03-0002; Dharmacon, Lafayette, CO, USA), and the respective miRIDIAN microRNA negative controls (mimic negative control number 1, CN-001000-01-05; hairpin inhibitor negative control number 1, IN-001005-01-05; Dharmacon, Lafayette, CO, USA) were combined with Opti-MEM reduced serum medium and RNAiMAX lipofectamine (Thermo Fisher Scientific, Waltham, MA, USA) to form a complex before transfection. This complex, carried with 30 nM of each oligonucleotide, was incubated for 15 h with C2C12 myotubes (Supplementary Figure S1B). All experiments were carried out using at least three independent replicates per group.

### 2.3. Immunofluorescence

C2C12 myotubes were cultured in 6-well plates, fixed in 4% paraformaldehyde for 15 min, washed with PBS containing 0.1% TritonX-100 (Sigma, St. Louis, MO, USA), and blocked with 3% BSA, 1% glycine, 8% fetal bovine serum in PBS for 1 h at room temperature. The cells were incubated with primary Myh2 antibody (SC-53094, 1:500 dilution) overnight at 4 °C and, after washing, incubated with secondary antibodies for 1 h at room temperature and counterstained with DAPI. All images were obtained at room temperature using the scanning confocal microscope TCS SP5 (Leica Microsystems, Wetzlar, Germany). The area and diameter of the myotubes were measured using NIH ImageJ software (https://imagej.nih.gov/ij/ accessed on 21 January 2019). Fifteen fields were randomly selected in each group, and at least 100 myotubes were analyzed. We determined the average diameter by calculating the mean of the measures taken along the myotubes’ length, as previously described by Rommel et al. [43].

### 2.4. EdU Assay

The 5-Ethynyl-2′-deoxyuridine (EdU) incorporation assay was performed using an EdU assay kit (Click-iT™ EdU Cell Proliferation Kit for Imaging—Thermo Fisher Scientific, Waltham, MA, USA) following the manufacturer’s instructions. EdU labeling was conducted for 4 h for the C2C12 myoblasts with miR-497-5p mimic or IL-6 (100 ng/mL) and the respective controls. The proportions of EdU- and DAPI-positive cells were measured using ImageJ software (https://imagej.nih.gov/ij/ accessed on 09 January 2019).

### 2.5. RNA Isolation

The total RNA was extracted from C2C12 cells using TRIzol reagent (Thermo Fisher Scientific, Waltham, MA, USA), as recommended by the manufacturer. RNA concentration and quality were assessed using a NanoVue Plus Spectrophotometer (GE Healthcare, Chicago, IL, USA). The RNA quality was also assured by the RNA integrity number (RIN) obtained by analyzing ribosomal RNAs based on microfluidics using the 2100 Bioanalyzer system (Agilent Technologies, Santa Clara, CA, USA). Only RNA samples with 260 nm/280 nm ratio of 1.8–2.0, 260 nm/230 nm ratio > 2.0, and RIN > 9 were used for subsequent analysis.

### 2.6. Global MicroRNA Expression Profiling

We used the TaqMan^®^ MicroRNA Reverse Transcription Kit and Megaplex™ RT Primers (Thermo Fisher Scientific, Waltham, MA, USA) to synthesize single-stranded cDNA from total RNA samples. We used a total of 350 ng of resulting cDNA to prepare a PCR mix by combining it with 50 μL TaqMan Gene Expression master mix (Thermo Fisher Scientific, Waltham, MA, USA) and water up to a final volume of 100 μL. Global microRNA profiling of 373 mature microRNAs from the control (*n* = 3) and IL-6 [100 ng/mL] treated (*n* = 3) myotubes was performed with the TaqMan^®^ Array Rodent MicroRNA Cards A v3.0 (Thermo Fisher Scientific, Waltham, MA, USA) following the manufacturer’s instructions. The samples were run on the QuantStudio™ 12K Flex System (Thermo Fisher Scientific, Waltham, MA, USA) using the following cycle conditions: 92 °C for 10 min followed by 40 cycles of 95 °C for 15 secs and 60 °C for 1 min. Small RNA MammU6 was used as a reference to normalize the microRNA data. Finally, the raw data from each set of cards were retrieved and imported into Expression Suite Software v1.0.3 (Thermo Fisher Scientific, Waltham, MA, USA). Relative quantification of microRNA expression was evaluated using the 2^−ΔΔCT^ method [44]. Cutoffs for significant changes were set at a fold-change of 1.5 and *p*-value ≤ 0.05.

### 2.7. mRNA Gene Expression

mRNA samples were reverse transcribed using the High-Capacity RNA-to-cDNA master mix (Thermo Fisher Scientific, Waltham, MA, USA). The mRNAs qPCR analysis was performed in a 15 µL reaction with Power SYBR™ Green master mix for mRNAs (Thermo Fisher Scientific, Waltham, MA, USA), as described by the manufacturer. The samples were run on QuantStudio™ 12K Flex System (Thermo Fisher Scientific, Waltham, MA, USA) using the following cycle conditions: 95 °C for 10 min, 40 cycles of 95 °C for 15 secs, and 60 °C for 1 min. The oligonucleotides used for the mRNA analyses are listed in Supplementary Table S1. *Rpl13a* was used as a reference gene to normalize the mRNA data. Relative quantification of mRNA expression was evaluated using the 2^−ΔΔCT^ method [44]. The reference gene was selected based on geNorm calculations [45]. The cutoffs for significant changes were set at a fold change of 1.5 and a *p*-value < 0.05.

### 2.8. Prediction of miRNA-Targets Genes and Gene Ontology and Pathway Enrichment Analysis

The deregulated miRNAs in myotubes treated with IL-6 were used to predict mRNAs targets using the target prediction algorithm TargetScan 7.1 [46], and the database miRTarBase [47] was used to confirm the potential interactions. Protein-protein interaction (PPI) networks were generated using Metasearch STRING v10.5.1 [48,49], and visualization and annotation data for the PPI and miRNA gene interaction networks were generated using Cytoscape v3.4.0 [50]. Gene Ontology and pathways for differentially expressed miRNAs were explored by EnrichR [51,52] (http://amp.pharm.mssm.edu/Enrichr/ accessed on 14 October 2019), and the enrichment result is represented by *p*-value (Fisher exact test) and Z-score (correction to the test) in a combined score computed by EnrichR.

### 2.9. Meta-Analysis of Cancer Cachexia Data Sets

To confirm whether the identified miR-497-5p targets are deregulated in cancer cachexia models, we compared publicly available muscle gene expression profiles with our gene expression data after inhibiting miR-497-5p in C2C12 myotubes. The gene expression profiling data used in the meta-analysis in the present study were accessed from the NCBI Gene Expression Omnibus (GEO): GSE48363 (colon adenocarcinoma) [53], GSE63032 (colon adenocarcinoma) [54], GSE51931 (pancreatic cancer) [24], and GSE24112 (colon adenocarcinoma) [25]. We re-analyzed each data set individually using GEO2R (http://www.nci.nlm.nih.gov/geo/geo2r/ accessed on 28 August 2019). GEO2R is an interactive, online tool for R-based analysis of GEO data to identify differentially expressed genes across experimental conditions [55]. We followed the limma-voom pipeline to identify DEGs in cancer cachexia samples. The list of deregulated genes from each dataset includes those with a fold-change of 2 and adjusted *p*-value < 0.05 when compared to control samples.

### 2.10. Statistical Analysis and Data Visualization

All experimental data were normally distributed and expressed as the mean ± standard deviation (SD). Data were analyzed using the Student’s *t*-test (GraphPad Prism 6), and differences with a *p*-value < 0.05 were considered significant. The Venn graph was created using the software Lucidchart (https://www.lucidchart.com/ accessed on July 2019). The heatmaps were created using the web tool Morpheus [56] (https://software.broadinstitute.org/morpheus accessed on August 2019).

## 3. Results

### 3.1. IL-6 Induces C2C12 Myotube Atrophy

**Figure 1:**
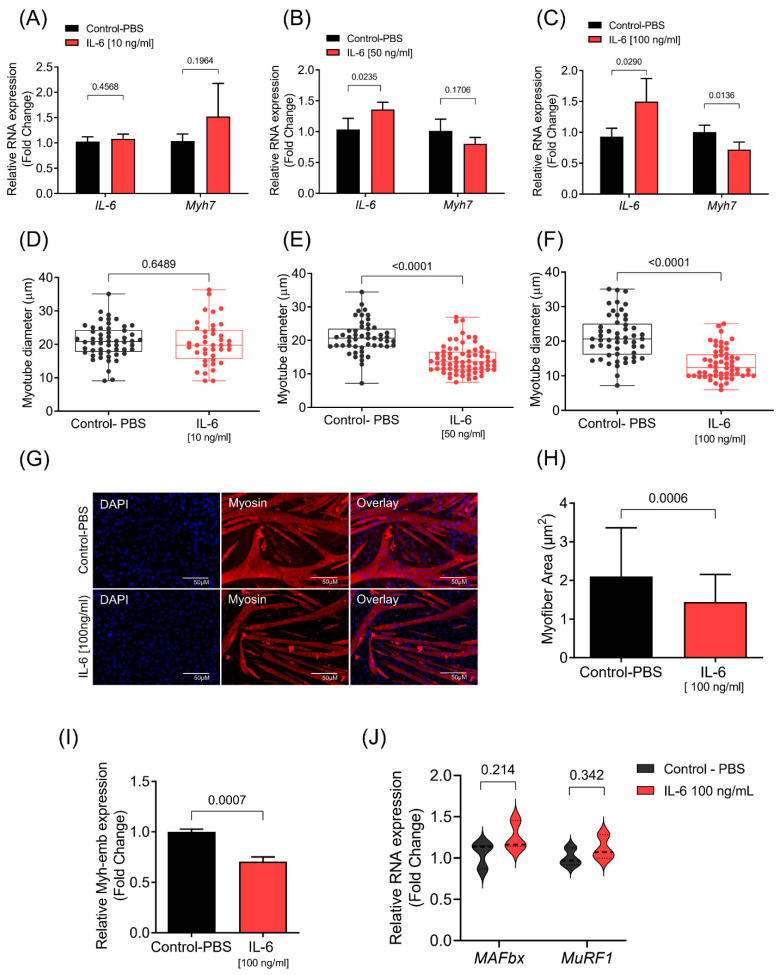
IL-6 induces C2C12 myotube atrophy. (**A**–**C**) IL-6 and Myh7 mRNA levels in differentiated C2C12 myotubes treated with three different concentrations of IL-6. RT-qPCR data are represented as fold change (2^ΔΔCt^) relative to *Rpl13a*. Data represent the average of three independent experiments with standard deviation. (**D**–**F**) Diameter of myotubes treated with IL-6 (**D**, 10 ng/mL; **E**, 50 ng/mL; **F**, 100 ng/mL) myotubes with their corresponding controls. (**G**), Representative images of myotubes from both control and IL-6 treated (100 ng/mL) myotubes. Immunofluorescence was performed using the antibody against Myh2 and Dapi (4′,6-Diamidine-2′-phenylindole dihydrochloride). (**H**) Quantitative analysis of the myotube area of control and IL-6 treated myotubes (100 ng/mL) myotubes. (**I**) mRNA levels of embryonic myosin (Myh-emb) and (**J**) mRNA levels of atrogenes *MAFbx* and *MuRF1* in differentiated C2C12 myotubes treated with IL-6 (100 ng/mL). RT-qPCR data are presented as fold change (2^−ΔΔCt^) relative to *Rpl13a*. Statistical difference was analyzed by Student’s *t*-test.

First, to test whether IL-6 could induce myotubes atrophy in vitro, we evaluated the effect of three different IL-6 concentrations (10 ng/mL, 50 ng/mL, and 100 ng/mL) on differentiated C2C12 myotubes. While differentiated myotubes treated with 50 ng/mL or 100 ng/mL exogenous IL-6 increased the transcription of endogenous IL-6, the presence of 10 ng/mL IL-6 did not affect the expression of the endogenous *Il6* gene (Figure 1A–C). The expression of *Myh7* did not change in myotubes treated with 10 ng/mL and 50 ng/mL IL-6, but it was decreased in myotubes treated with 100 ng/mL of IL-6 (Figure 1A–C). Consistent with endogenous expression of IL-6, 10 ng/mL IL-6 did not reduce the diameters of the myotubes (Figure 1D), while 50 ng/mL and 100 ng/mL IL-6 indeed significantly reduced both the diameter and area of the myotubes (Figure 1E–H). Furthermore, 100 ng/mL IL-6 decreased the expression of embryonic myosin (Myh-emb), a typical and abundant myosin isoform of C2C12 myotubes (Figure 1I). Furthermore, the increased concentrations of IL-6 resulted in a high percentage of small myotubes (Figure S2). The levels of the atrogenes *MAFbx* and *MuRF1* did not differ between the groups (Figure 1J), suggesting that additional signaling pathways, independent of *MuRF1* and *MAFbx* expression, are also involved in muscle atrophy. This result is in line with previous studies showing that signaling networks that control the atrophy program are specific for each catabolic condition [15,36,57]. Together, our data showed that IL-6 induces myotube atrophy at high concentrations (100 ng/mL).

### 3.2. Global miRNA Expression Analysis Identified miR-497-5p as a miRNA Involved in Muscle Atrophy

**Figure 2:**
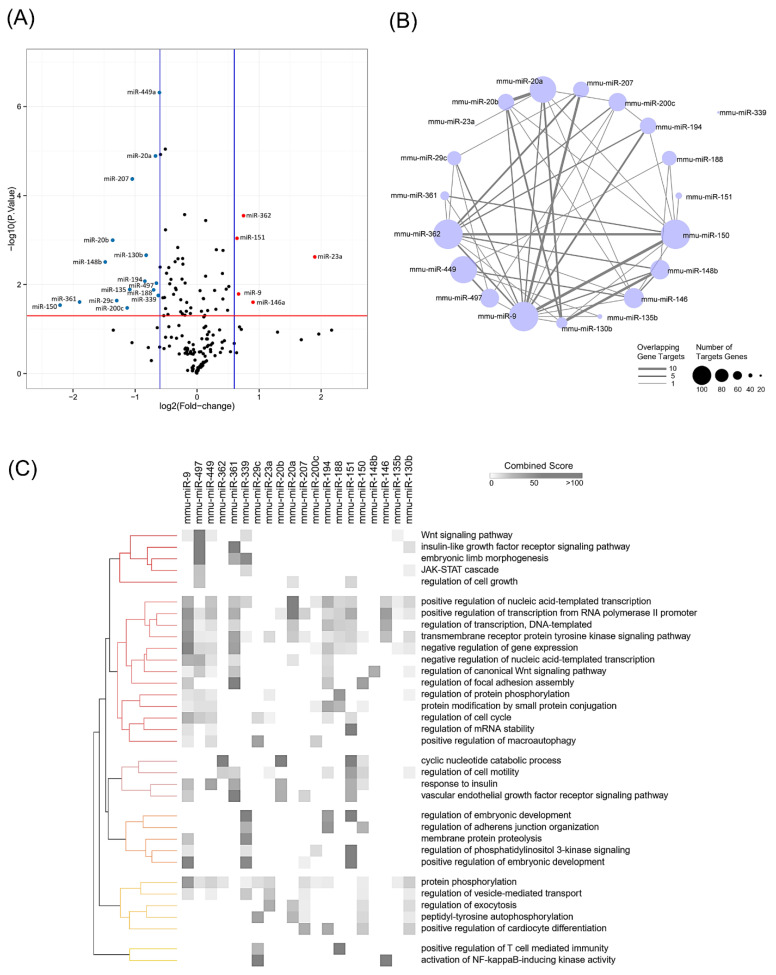
Global miRNA expression in IL-6-treated myotubes. (**A**) Volcano plot representing the log 2 fold change and *p*-value of all microRNAs differentially expressed. A set of 20 miRNAs displayed significant changes (*p*-value < 0.05 and fold change ≥ 1.5). The blue and red dots represent the down- and up-regulated microRNAs, respectively. (**B**) Interaction network representing the 100 interactions between 19 miRNAs and 116 predicted target genes. Node size indicates the number of the miRNA-target gene, and the gray edge width denotes overlapping miRNA-target gene transcripts. (**C**) Heatmap of the biological processes (GO) enriched by a predicted set of targets for each miRNA. The top five ranked annotations of GO and enriched in at least two datasets were included in the heatmap. Unsupervised clustering was performed according to the Kendall rank correlation coefficient.

Twenty miRNAs, five up-regulated and 15 down-regulated, were differentially expressed in myotubes treated with IL-6 (100 ng/mL) in our global miRNA expression profiling analysis (Figure 2A and Supplementary Table S2). The list with all miRNA expression data was presented in Supplementary Table S3. This analysis identified some miRNAs previously associated with skeletal muscle atrophy, such as miR-23a, miR-146a, and miR-29c [58,59,60]. Next, we predicted the targets for each identified miRNA. To better understand the miRNAs interactions in IL-6 induced atrophy, we predicted common targets among the 20 dysregulated miRNAs. We found a network with 100 interactions between 19 miRNAs and 116 target genes (Figure 2B). The over-represented Gene Ontology categories (biological processes) for these targets included target genes regulating cyclic nucleotide catabolic process, activation of NFκB-inducing kinase activity, insulin-like growth factor signaling, Janus kinase/signal transducers and activators of transcription (JAK-STAT) cascade, and regulation of cell cycle (Figure 2C). These results indicate a complex combination of target multiplicity and miRNA cooperativeness on muscle plasticity induced by IL-6.

**Figure 3:**
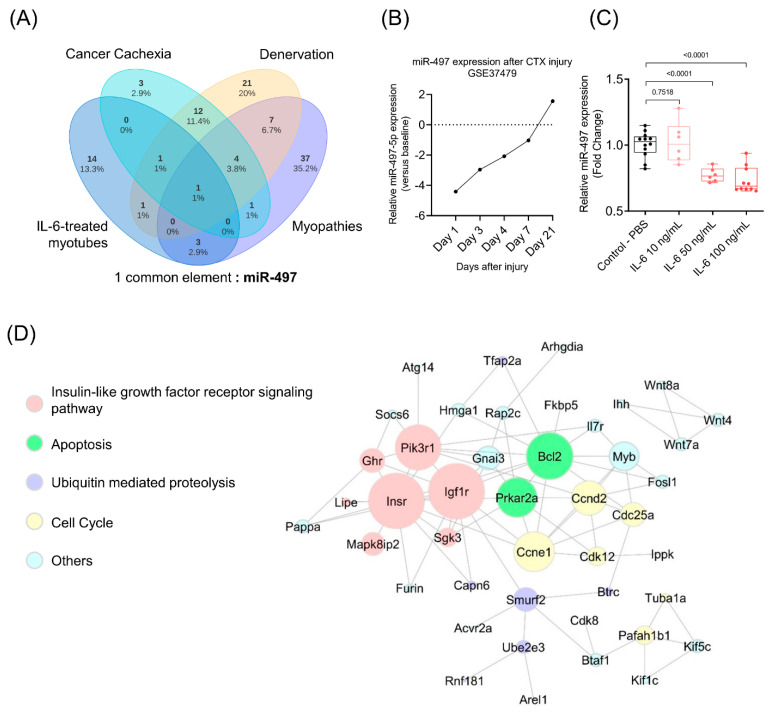
miR-497-5p is deregulated in skeletal muscle cells in different atrophic conditions. (**A**) Venn diagram showing the miR-497-5p as a common element between our data, cancer cachexia, primary muscle disorders, and other catabolic conditions. (**B**) miR-497 expression levels after 1, 3, 4, 7, and 21 days of cardiotoxin injury (CTX). Relative miRNA expression versus baseline (GSE37479). (**C**) MiR-497-5p expression levels in differentiated C2C12 myotubes treated with three different concentrations of IL-6 vs. control (PBS). RT-qPCR data are presented as fold change (2^−ΔΔCt^) relative to MammU6. (**D**) Interactome between the predicted miR-497-5p target genes. Nodes represent miR-497-5p target genes according to the biological process. The larger the nodes, the higher the degree of interactions identified. Nodes with no connections were excluded. STRING v10.5.1 was used to generate protein interactions, and the network interactions were visualized using Cytoscape v3.4.0.

We compared our list of deregulated miRNAs with previous studies that reported alterations in miRNA expression under muscle atrophy conditions [35,36]. In our analysis, we included experimental models of cancer cachexia, denervation, and expression data from patients with primary disorders [35,36]. The miRNA expression profile was obtained from mice tibialis anterior muscle 14 days after denervation [36] or with cancer cachexia (colon adenocarcinoma c26 model) [36]. We also included the list of microRNA profiles obtained from muscle specimens of patients with 11 primary muscle disorders (Becker muscular dystrophy, dermatomyositis, Duchenne muscular dystrophy, facioscapulohumeral muscular dystrophy, polymyositis, limb-girdle muscular dystrophy types 2A and 2B, Miyoshi myopathy, nemaline myopathy, and polymyositis) [35]. This comparison revealed that the miRNAs miR-23a-5p and miR-497-5p were deregulated in cancer cachexia [36]; miR-146a-5p, miR-151-3p, and miR-497-5p in primary disorders [35], and miR-23a-5p, miR-29c-5p, miR-449a-3p, and miR-497-5p in different catabolic conditions [36] (Figure 3A). Most interestingly, we identified miR-497 as differentially expressed in all conditions analyzed. Furthermore, to explore the potential of miR-497-5p in muscle regeneration, we sought to analyze its expression in muscles from cardiotoxin-injured mice. We collected relative miRNA expression data for muscle cardiotoxin injury (CTX injury) in mice available in Chen et al. 2012 [61] (GSE37479). These results show decreased expression of miR-497 after day 1, with a subsequent increase after 3 and 4 days of CTX-injury (Figure 3B). We validated by RT-qPCR that the miR-497-5p is indeed down-regulated in myotubes treated with high concentrations of IL-6 (50 ng/mL and 100 ng/mL) (Figure 3B). To elucidate the post-transcriptional mechanisms of the miR-497-5p that could potentially affect the myotubes’ size, the targets of this miRNA were predicted using TargetScan 7.2 (http://www.targetscan.org/ accessed on 15 October 2019). Interestingly, miR-497-5p did not affect the expression of myokines that are induced by IL6. The miR-497-5p targets interaction network shows several of its targets involved in the insulin signaling pathway, cell cycle, ubiquitin-mediated proteolysis, and apoptosis. The complex interactome analysis of miR-497-5p target genes and their respective functional annotations are illustrated in Figure 3C.

### 3.3. MiR-497-5p Overexpression Induces Myotube Atrophy

**Figure 4:**
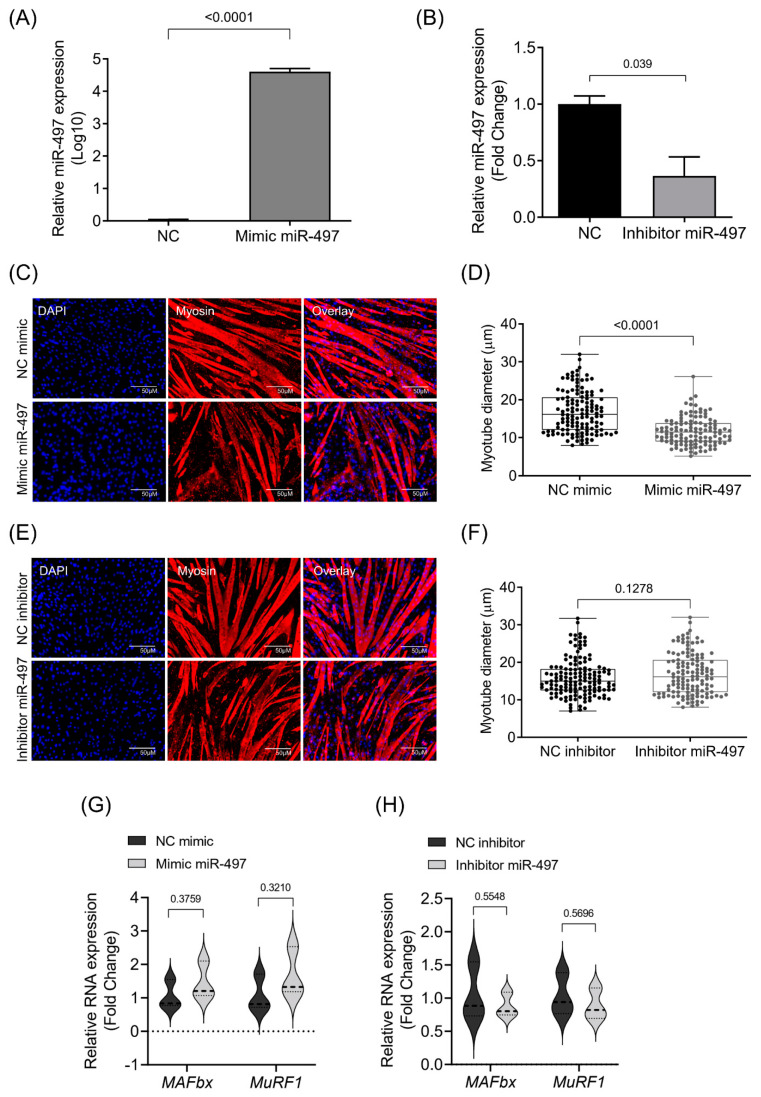
miR-497-5p effects in differentiated C2C12 myotubes. (**A**) Relative log10 transformed miR-497-5p levels measured by RT-qPCR in C2C12 myotubes transfected with mimic miR-497-5p, compared to C2C12 myotubes transfected with mimic control. (**B**) RT-qPCR showing decreased expression of miR-497 in C2C12 myotubes transfected with miRNA inhibitor against miR-497-5p. (**C**) Representative images of myotubes transfected with negative control or with miR-497-5p mimic. The immunofluorescence was performed using the antibody against Myh2 (red) and Dapi (blue). (**D**) Diameter of myotubes transfected with negative control or miR-497-5p mimic. (**E**) Representative images of myotubes from the negative control or transfected with miR-497-5p inhibitor. (**F**) Diameter of myotubes transfected with negative control or miR-497-5p inhibitor. (**G**), mRNA levels of atrogenes *MAFbx* and *MuRF1* C2C12 myotubes transfected with mimic miR-497-5p and (**H**) in C2C12 myotubes transfected with miRNA inhibitor against miR-497-5p. Statistical difference was analyzed by Student’s *t*-test.

Considering that IL-6 affects the miR-497-5p level in C2C12 myotubes, we next asked whether miR-497-5p may induce atrophy of skeletal muscle cells. RT-qPCR confirmed the efficiency of miRNA mimics or inhibits miR-497-5p expression in myotubes (Figure 4A,B). Previous reports suggest that this miRNA is involved in atrophic conditions, such as cancer cachexia [31], denervation [36], and myopathies [35]. Consistent with these findings, we observed the miR-497-5p mimic induced atrophy of C2C12 myotubes (Figure 4C,D). We next examined whether changes in the myotube size accompany the overexpression or inhibition of miR-497-5p. We found significant differences in gene expression for some regulators of myotube growth. Myogenin (*Myog*) expression was increased in myotubes overexpressing miR-497-5p, but no differences were observed in myotubes transfected with miR-497-5p inhibitors (Suppl. Figure S4). Furthermore, we investigated the expression of the *Myh7*, *Hmga1*, and *Id3* genes. No differences were observed in the expression of *Myh7* and *Id3* after the treatment of the cells with miR-497-5p mimics or inhibitors. However, the expression of the *Hmga1* gene was down-regulated in myotubes transfected with the miR-497-5p mimic and inhibitor (Figure S4). Immunofluorescence analysis revealed that the transfection with miR-497-5p mimic decreased myotube diameter (Figure 4C,D) while the miR-497-5p inhibitor did not change myotube size (Figure 4E,F). Thus, it seems that miR-497-5p targets genes that regulate muscle atrophy or that this miRNA controls muscle cell homeostasis in some way that needs further exploration.

### 3.4. The Insulin Signaling Pathway Is Enriched with miR-497-5p Targets Genes

**Figure 5:**
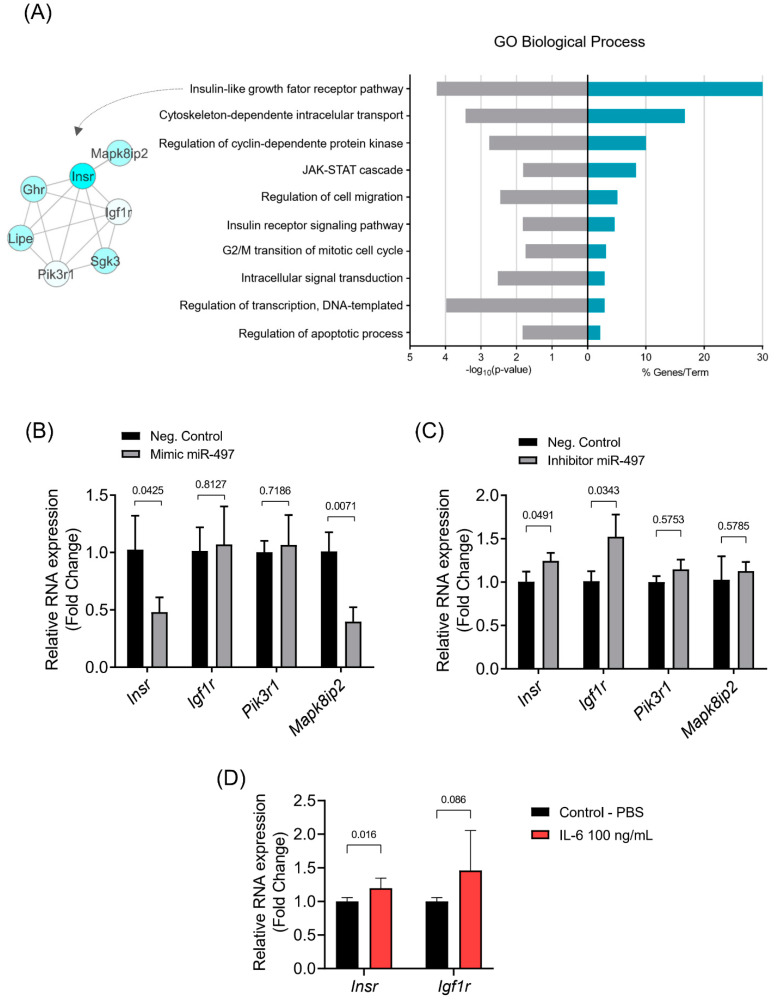
miR-497-5p target genes enrich the insulin-growth factor pathway. (**A**) Biological processes terms (GO) enriched for miR-497-5p target genes (*p*-value ≤ 1.82 × 10^−2^). *Insr*, *Igf1r*, *Pik3r1*, and *Mapk8ip2* mRNA levels in differentiated C2C12 myotubes transfected with miR-497-5p mimic (**B**) or inhibitor (**C**). (**D**) *Insr* and *Ingf1r* expression levels in differentiated C2C12 myotubes treated with IL-6 and control (PBS). RT-qPCR data are represented as fold change (2^−ΔΔCt^) relative to *Rpl13a*. Data represent the average of three independent experiments with standard deviation. Statistical difference was analyzed by Student’s *t*-test. *Insr*: insulin receptor; *Igf1r*: insulin-like growth factor 1 receptor; *Pik3r1*: phosphoinositide-3-kinase regulatory subunit 1; *Mapk8ip2*: mitogen-activated protein kinase 8 interacting protein 2.

We sought to identify the biological functions of miR-497-5p targets in skeletal muscle cells. According to the GO analysis, the list of miR-497-5p target genes highlights the insulin-growth factor receptor pathway as the most overrepresented (enriched) biological process (Figure 5A). Moreover, through other gene set enrichment analysis databases (KEGG, WikiPathways, and GO molecular function), we identified several additional enriched terms represented by the miR-497-5p target genes (Figure S3). We found that ubiquitin-mediated proteolysis, cell cycle, apoptosis, and the JAK-STAT signaling pathway are overrepresented in miR-497-5p target genes. The insulin pathway is enriched in the biological process and WikiPathways databases (Figure 5A and Figure S4). Among the genes that enriched the insulin-signaling pathway, *Insr*, *Igf1r*, *Pik3r1*, and *Mapk8ip2* genes were further analyzed by RT-qPCR. Myotubes transfected with mimic miR-497-5p decreased the expression of *Insr* and *Mapk8ip2*, while the miR-497-5p inhibitor increased the expression of *Insr* and *Igf1r.* Neither the miR-497-5p mimic nor its inhibitor affected the expression of *Pik3r1* (Figure 5B,C). The increased expression in *Insr* and *Igf1r* induced by the mir-497-5p inhibitor led us to investigate the possibility of a feedback loop that counteracts IL-6-induced muscle atrophy. We observed that IL-6 treatment decreases the expression of *Insr* but not *Igf1r* (Figure 5D). Together, these results suggest that miR-497-5p regulates genes in the insulin-growth factor receptor pathway, which may play a compensatory mechanism during IL-6-induced skeletal muscle atrophy.

### 3.5. Cellular Proliferation Rate Is Not Affected by IL-6 or miR-497-5p

**Figure 6:**
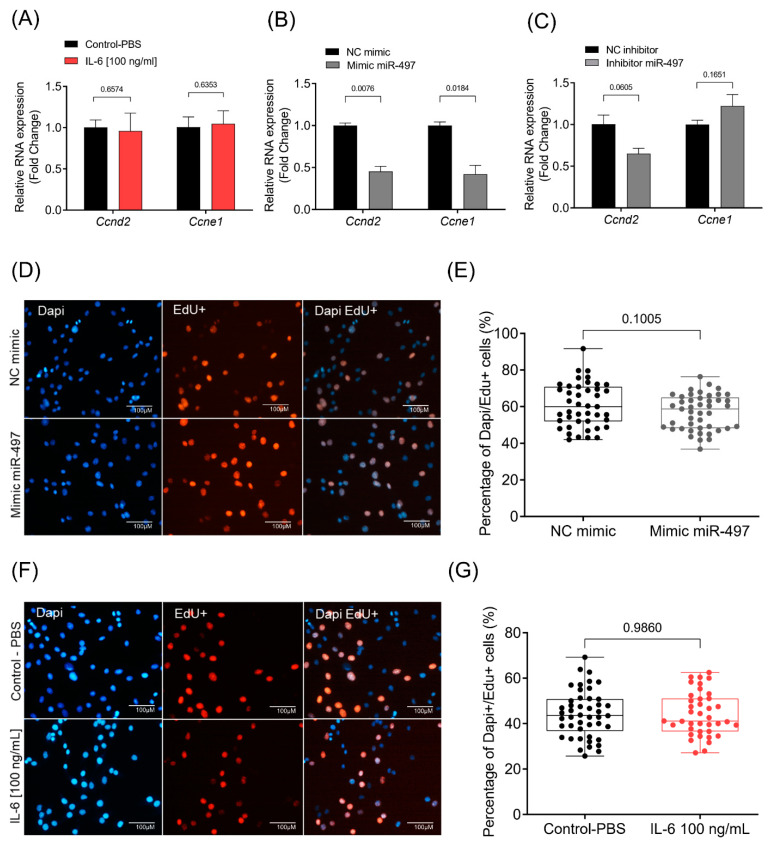
miR-497-5p and cellular proliferation. mRNA levels of the cyclins *CcnD2* and *CcnE1* in C2C12 myoblasts treated with IL-6 [100 ng/mL] or PBS (**A**) transfected with miR-497-5p mimic or negative control (**B**) and transfected with miR-497-5p inhibitor or negative control. (**C**) RT-qPCR data are presented as fold change (2^−ΔΔCt^) relative to *Rpl13a*. (**D**) Representative images of myoblasts transfected with miR-497-5p mimic or negative control. Cells were stained with EdU (red). (**E**) Percentage of Dapi (blue), and Edu+ cells from the total Dapi cells. (**F**) Representative images of myoblasts treated with IL-6 (100 ng/mL) or PBS (control) and stained with EdU (red). (**G**) Percentage of Dapi (blue), and Edu+ cells out of the total Dapi cells. Data represent the average of three independent experiments with standard deviation. Statistical difference was analyzed by Student’s *t*-test.

The term cell cycle also appeared among the pathways enriched in our ontology analysis. The mir-497-5p has already been reported to play an essential role in regulating skeletal muscle cell proliferation [62]. Cyclins *CcnD2* and *CcnE1* have been predicted as miR-497-5p targets, and there is evidence that miR-497-5p acts on muscle cell proliferation by regulating *CcnD2* and *CcnE1* [62]. The transfection of miR-497-5p mimic indeed significantly decreased the expression of *CcnD2* and *CcnE1*, while the inhibition of miR-497-5p and IL-6 treatment showed no change in the expression levels of these cyclins (Figure 6A–C). We sought to evaluate cell proliferation using an EdU assay to test whether myoblasts had impaired proliferation after being treated with miR-497-5p mimic or IL-6. Surprisingly, we found no alteration in myoblasts proliferation rate neither by miR-497-5p mimic nor by IL-6 treatment (Figure 6D–G). Thus, our results suggest that miR-497-5p regulates *CcnD2* and *CcnE1* expression but does not affect myoblast proliferation rate.

### 3.6. In Silico Validation Using Cancer Cachexia Models

**Figure 7:**
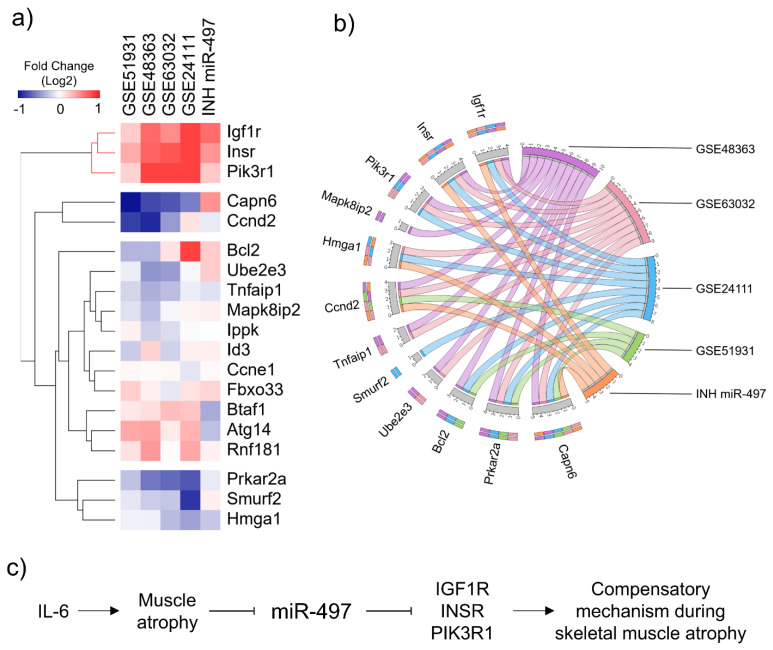
miR-497-5p involves the regulation of *Igf1r* and *Insr* genes and the activation of a compensatory molecular mechanism potentially able to reduce cell muscle wasting. (**a**) Heatmap of the expression levels (log2 fold change) of miR-497-5p target genes in four different cancer cachexia datasets and myotubes transfected with inhibitor miR-497-5p. Both rows (target genes) and columns (datasets) were clustered using the Kendall rank correlation coefficient. (**b**) Circos plot showing the overlapping genes among the studies. (**c**) Schematic diagram of the IL-6–miR-497–Igf1r/Insr pathway and its role in a compensatory mechanism during skeletal muscle atrophy.

We performed a meta-analysis of four muscle transcriptomic datasets of cachectic animal models available at NCBI Gene Expression Omnibus (GEO) to verify how consistent our findings are with those observed in cancer cachexia models. The expression profile of the miR-497-5p targets genes is shown in the heatmap (Figure 7a) and the bar graphs (Figure S4). Notably, the miR-497-5p-treated myotubes showed expression profiles of the *Igf1r* and *Insr* similar to those found in muscle samples from cachectic mice (Figure 7a). However, the expression pattern of several miR-497 targets varied between the studies (Figure 7b). These results indicate that the miR-497–Igf1r-Insr axis is consistently altered in different cachexia models and suggest that the imbalance between catabolism and anabolism may be accompanied by a compensatory molecular mechanism able to reduce cell muscle wasting (Figure 7b).

## 4. Discussion

Although many studies have shown the involvement of IL-6 in skeletal muscle atrophy due to systemic inflammation in cancer patients, little is known about the intracellular mechanisms regulated by this cytokine in skeletal muscle cells. The present study identified 20 miRNAs that are dysregulated during muscle cell atrophy induced by high concentrations of IL-6 in vitro. These miRNAs post-transcriptionally regulate genes involved in activating NFkappaB-inducing kinase activity, insulin-like growth factor signaling, JAK-STAT cascade, and cell cycle. Among them, miR-497-5p appeared to play a pivotal role in muscle wasting. Our meta-analysis confirmed that miR-497-5p is down-regulated in other atrophic conditions such as cancer cachexia, denervation, and myopathies. Our results also showed that the IL-6-induced decrease in miR-497-5p expression is associated with the regulation of the insulin pathway genes *Insr* and *Igf1r.* Thus, our results reveal a compensatory molecular mechanism that may counteract muscle cell atrophy.

Several studies have indicated IL-6 with a critical regulatory role in skeletal muscle plasticity [34,63]. However, the effects of this cytokine in the muscle are paradoxes. This divergence between catabolism or anabolism IL-6-mediated effects is associated with its acute or chronic exposures and the type of administration [18]. Therefore, we mimicked IL-6 chronic exposure and tested the impact of different cytokine concentrations on C2C12 myotubes to understand the role of this cytokine on muscle atrophy. Our results showed that a supraphysiological dose of 100 ng/mL could be preferentially used to induce catabolic effects in myotubes. These results are consistent with a previous report that tested 10 ng/mL and 100 ng/mL in this same cell line and found that myotubes treated with 100 ng/mL of IL-6 decrease the myotubes diameter by 36% [12].

We also found that this supraphysiological dose of IL-6 (100ng/mL) leads to myotube atrophy and deregulates the expression of 20 miRNAs. Our prediction of miRNA-target genes revealed several biological processes potentially post-transcriptional regulated by changes in miRNA expression induced by high IL-6 exposure. These biological processes include activation of insulin-like growth factor receptor pathway, NFκB-inducing kinase activity, JAK-STAT cascade, regulation of T cell-mediated immunity, and cell cycle. Our enrichment results agree with recent transcriptome data obtained in mice atrophying muscles after infusion of IL-6 [34]. Further, to understand which miRNA could be crucial during the IL-6-induced atrophy, we performed a comparative analysis that identified miR-23a-5p and miR-497-5p also deregulated in skeletal muscle of cancer cachexia models [36]. However, the directions of the expression changes for miR-23a-3p differed between our studies and previous cachexia studies. Soares et al. demonstrated that miR-23a-5p is down-regulated in a mouse model of cachexia-induced muscle wasting, while miR-23a-5p levels increased in our IL-6-induced atrophied myotubes. Several other studies have pointed out that miR-23a-5p is an important miRNA involved in muscle plasticity (reviewed by Wang [64]). For instance, some studies showed an increase in miR-23a-5p expression associated with muscle atrophy, suggesting that miR-23a-5p is a critical regulatory factor in denervation-induced skeletal muscle atrophy [65,66]. On the other hand, studies also point out that increased expression of miR-23a-5p is essential to attenuate muscle loss [67,68]. Despite these differences in miR-23a-5p expression pattern in models, when we compared miRNAs datasets across different atrophy conditions, the levels of miR-497-5p were consistently reduced in our experiments as well as in cancer cachexia and denervation-induced atrophy models [36].

There is consistent evidence showing that miR-497-5p is a crucial player in skeletal muscle development [62,69]. Previous studies have reported a regulatory axis for myogenesis in which miR-195/497 promotes myogenic proliferation and differentiation by repressing the HMGA1–Id3 pathway [69]. To validate this role of miR-497-5p in proliferation, we performed in vitro assays to confirm this antiproliferative effect on myoblasts. Although miR-497-5p affected the expression of *Hmga1*, we observed that miR-497-5p neither changed the *Id3* expression (Figure S4) nor the proliferation rate of myoblasts. These discrepancies may be related to the different protocols used to evaluate proliferation. Another study showed that higher levels of miR-497-5p decrease the expression of cyclins *Ccnd2* and *CcnE1*, leading to a reduction in myoblast proliferation [62]. We observed that miR-497-5p indeed inhibited the expression of these cyclins; however, our functional studies do not corroborate the reduction in cellular proliferation found by these authors.

Although the above studies have already shown the role of miR-497-5p during skeletal muscle development, the mechanisms by which miR-497-5p acts during muscle cell atrophy are mainly unknown. We analyzed the overexpression or inhibition of miR-497-5p in differentiated myotubes. The inhibition of miR-497-5p did not affect the myotubes size. This result might mean that IL-6 has additional mechanisms that induce muscle cell atrophy or control muscle cell size. Furthermore, miR-497-5p overexpression decreased myotube size, confirming that this miR-497-5p is associated with muscle atrophy or with control of cell size.

Additionally, the overexpression of miR-497-5p decreased in myotubes size, indicating that the high expression levels of miR-497-5p may not be beneficial to differentiated myotubes. Our findings also suggest that miR-497-5p regulates essential genes of the insulin-like growth factor (IGF) pathway, such as *Igf1r* and *Insr*. Several different signaling pathways control muscle mass. The insulin-like growth factor is a positive signaling pathway, as it increases muscle mass upon increasing protein synthesis and decreasing protein degradation [70,71]. Although miR-497-5p targets of the IGF signaling pathway have already been validated by other authors [62], a limitation of our report that needs further investigation is the lack of a protein analysis of IGF1R and INSR. Furthermore, any functional analysis regarding the interaction of miR-497-5p and these targets remains to be investigated.

Although it is possible that other pathways are affected by the miR-497-5p, the overexpression of growth-related genes was consistent with the results of our reanalysis of cancer cachexia datasets [24,25,53,54]. The proteins IGF1, INSR, and PI3KR1 play an essential role in the induction of skeletal muscle hypertrophy by increasing protein synthesis and blocking protein degradation [62,63]. Despite being intriguing, the high expression of these factors in the context of atrophy is reasonable when considering that muscle strives to maintain muscle homeostasis, even during atrophy conditions. By activating the expression of *Insr*, *Igf1*, and *Pik3r1*, the anabolic pathway is activated to balance the catabolic process. Thus, inhibition of miR-497-5p after IL-6 treatment, with consequent increased expression of *Igf1* and *Insr*, may suggest a compensatory molecular mechanism involving miR-497-5p during IL-6-induced muscle cell atrophy.

In summary, we first found that IL-6 induces atrophy and decreases miR-497-5p levels. Secondly, we observed that the expression of miR-497-5p is reduced under atrophic conditions. Thirdly, the miR-497 targets the genes *Igf1r*, *Insr*, and *Pik3r1*, which are known to induce skeletal muscle hypertrophy [72]. Surprisingly, we found that these genes associated with hypertrophy are highly expressed during muscle wasting in cancer cachexia. This high expression of genes associated with hypertrophy may constitute a mechanism that counteracts atrophy under the control of miR-497-5p. A plausible explanation for this mechanism is that miR-497-5p and the target genes *Igf1r* and *Insr* may be associated with muscle cell homeostasis or boosting antiatrophic processes. Consequently, the high levels of miR-497-5p inhibited these hypertrophy genes and resulted in the atrophy of the C2C12 myotubes. In conclusion, IL-6 induces atrophy and decreases miR-497-5p as a feedback loop that increases the expression of genes associated with hypertrophy to counteract atrophy. Our findings provide insights into new mechanisms during muscle atrophy and identify miR-497-5p as a potential pharmacological target for cancer cachexia treatment.

## Data Availability

The published transcriptome datasets can be found in the NCBI Gene Expression Omnibus (GEO) database https://www.ncbi.nlm.nih.gov/gds (IDs: GSE48363, GSE63032, GSE51931, GSE24112, and GSE37479).

## Supplementary Materials

The following are available online at https://www.mdpi.com/article/10.3390/cells10123527/s1, Figure S1: Experimental design of cell culture, Figure S2: Effect of three different IL-6 concentrations on differentiated C2C12 myotubes, Figure S3: Enrichment analysis overrepresented by the miR-497-5p target genes, Figure S4: Expression profile of miR-497-5p target genes, Table S1: Primers for the expression analysis by RT-qPCR of the mRNAs indicated, Table S2: Differentially expressed microRNAs in skeletal muscle cells treated with IL-6 ranked by a combination of *p*-value < 0.05 and fold change ≥ 1.5, Table S3: Taqman low density array_expression raw data_Ct (threshold cycle).
